# The effect of moving cupping on psoriasis vulgaris and its influence on PASI score: A protocol for systematic review and meta-analysis

**DOI:** 10.1097/MD.0000000000024217

**Published:** 2021-02-12

**Authors:** Wu Yunbo, Liu Mingqiang, Qiu Guirong, Lan Hongrong, Huang Gang

**Affiliations:** a Affiliated Hospital of Jiangxi University of Traditional Chinese Medicine; b Jiangxi University of traditional Chinese medicine, 56 Yangming Road, Donghu District, Nanchang City, Jiangxi Province, PR China.

**Keywords:** meta-analysis protocol, moving cupping, psoriasis vulgaris

## Abstract

**Background:**

We aim to study the treatment of psoriasis vulgaris with moving cupping.

**Methods:**

We will search PubMed, Embase, the Cochrane Library, the China National Knowledge Infrastructure, Chinese Science and Technology Periodical Database, Wanfang Database, and Chinese Biomedical Literature Database of randomized controlled trials beginning from their inception to August 2020. The primary outcomes are that PASI score and clinical effective rate will be the main outcome indicators. Additional outcome is The Quality of life index score and safety assessment will be considered a secondary outcome. Two independent authors will based on the Cochrane system evaluation manual 5.1.0 version of RCT bias risk assessment tool to evaluate the risk of bias among the final included studies. And we will use the RevMan 5.3 software to analysis data.

**Results:**

This study will provide an assessment of the current state of moving cupping for the psoriasis vulgaris, aiming to show the efficacy and safety of this treatment.

**Conclusion:**

This study will provide evidence to judge whether moving cupping is an effective therapy for psoriasis vulgaris.

**Inplasy registration number:**

INPLASY2020120061.

## 1 Introduction

Psoriasis is a chronic skin disease with plaques and scales as the main manifestations. Its main pathogenesis is excessive epidermal thickening and immune response.^[1,2]^ Psoriasis is a common disease in dermatology. The prevalence of psoriasis is about 2% to 5% in the world.^[3]^ According to previous epidemiological reports, the incidence rate of this disease has increased from 0.123% to 0.470% ^[4]^ in Han population of China. At the same time, the disease is susceptible to genetic, infection, social pressure, immune and other factors are closely related.^[5]^ The long course of disease and the hard to subside rash are also increasing the psychological pressure of patients with psoriasis. In recent years, the treatment of psoriasis in traditional Chinese medicine has made great progress. More and more safe and effective external treatment methods of traditional Chinese medicine have been discovered. Among them, walking cupping therapy is favored by more and more doctors. Walking cupping therapy can effectively reduce the skin lesions of patients, however, due to the lack of systematic evaluation, the authors conducted a meta-analysis on RCT in the treatment of psoriasis vulgaris to provide further data support for clinical application.

## 2 General information and methods

### 2.1 Types of study

All randomized controlled clinical trial use moving cupping in the treatment of psoriasis vulgaris. There is no limit to the language of documents.

#### 2.1.1 Types of participants

All the patients included in the literature were diagnosed as psoriasis vulgaris, regardless of age, gender and nationality.

#### 2.1.2 Types of interventions

##### 2.1.2.1 Experimental interventions

The experimental group only used the method of cupping, and there was no limit to the medium, time and course of treatment.

##### 2.1.2.2 Control interventions

The control group was given conventional treatment, including drug therapy, NB-UVB, 308 nm excimer laser, etc., but the control group could not use cupping therapy.

#### 2.1.3 Types of outcome measures

##### 2.1.3.1 Primary outcomes

PASI score and clinical effective rate will be the main outcome indicators.

##### 2.1.3.2 Additional outcomes

Quality of life index score and safety assessment will be considered a secondary outcome.

### 2.2 Search methods for the identification of studies

#### 2.2.1 Electronics searches

We will choose search these electronic databases which contain PubMed, Embase, the Cochrane Library, the China National Knowledge Infrastructure, Chinese Science and Technology Periodical Database, Wanfang Database and Chinese Biomedical Literature Database. The time range of searching literatures is from the establishment of the database to August 2020. Search terms consist of disease (psoriasis, Psoriasis Vulgaris) and intervention (moving cupping, Cupping therapy, Push can) and research types (randomized controlled trial, controlled clinical trial, random trials). The PubMed search strategy is shown in Table 1.

**Table 1:** Search strategy used in PubMed database.

Number	Searching item
1	Psoriasis
2	Psoriasis Vulgaris
3	1 or 2
4	Moving cupping
5	Cupping therapy
6	Push can
7	4 or 5 or 6
8	randomized controlled trial
9	controlled clinical trial
10	random trials
11	8 or 9 or 10
12	3 and 7 and 11

#### 2.2.2 Search for other resources

We will also retrieve the relevant conference papers, and search for new trials related to fire needle of vitiligo on the WHO International Clinical Trials Registration Platform (ICTRP) and the Clinical Trials.gov.

### 2.3 Data collection and analysis

#### 2.3.1 Selection of studies

**Figure 1:**
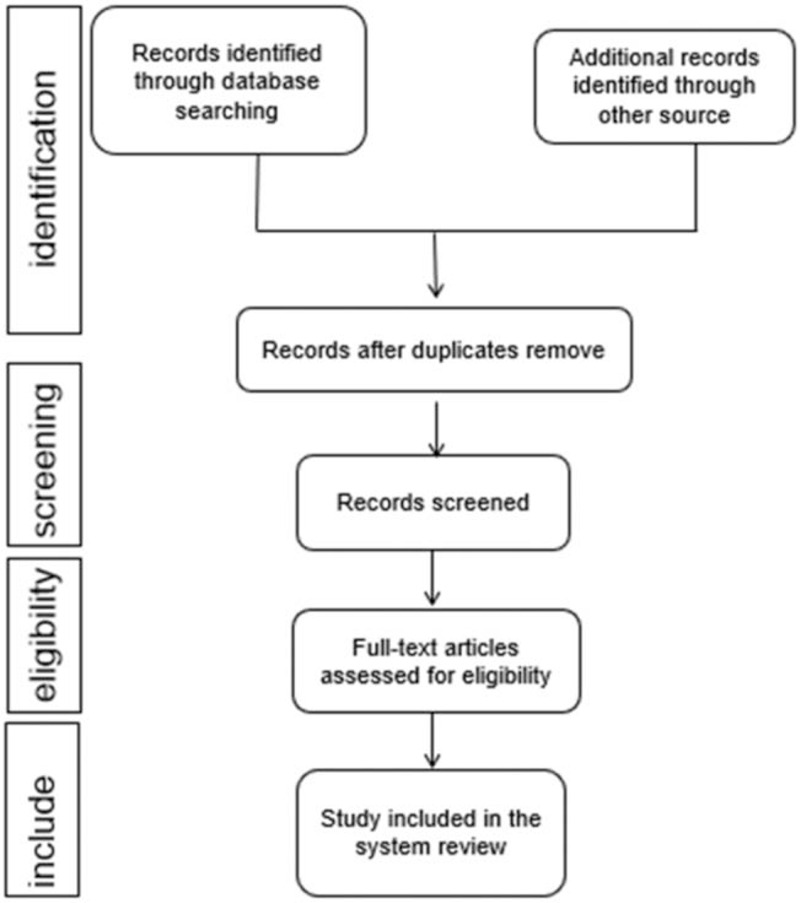
Flow diagram of study selection process.

We will import the retrieved literature into endnote x9 software, delete the duplicate files, and then 2 researchers independently read and screen the title, abstract and full text of the article to remove irrelevant articles. At the same time, the 2 researchers will select the appropriate literature according to the inclusion criteria, and will discuss if there are differences in the process. The study selection procedure is shown in Figure 1.

#### 2.3.2 Data extraction and management

Two researchers will independently extract the data, including the title of the literature, the author, the publication time, the number of cases in the literature, the patient's age, course of disease, intervention methods, outcome indicators, adverse reactions, dropped cases, etc. If necessary, we will try to contact the author for the details by email.

### 2.4 Risk of bias assessment

Two independent authors will based on the Cochrane system evaluation manual 5.1.0 version of RCT bias risk assessment tool^[6]^ to evaluate the risk of bias among the final included studies, the evaluation contents include:

1. randomized controlled trials;
2. whether allocation concealment is implemented;
3. whether the experiment is blind;
4. whether the evaluation of outcome indicators is blind;
5. whether the outcome indicators are complete;
6. whether selective reporting;
7. whether there are other bias risks.

When there are differences, the third researcher will negotiate and unify.

### 2.5 Quantitative data synthesis and statistical methods

#### 2.5.1 Quantitative data synthesis

Data analysis was performed using Revman 5.3 software provided by Cochrane. The measurement data (continuous variables) were expressed by weighted mean difference (WMD) or standard mean difference (MD) and 95% confidence interval (CI), while the count data (secondary variables) were expressed by odds ratio (or) and 95% confidence interval (CI). If *I*^2^ > 50%, it is considered that there is statistical heterogeneity, the random effect model is selected, otherwise, the fixed effect model is selected.

#### 2.5.2 Assessment of reporting biases

When more than 10 studies are included, funnel plot will be generated to detect the reporting bias. In addition, we will use the Egger test to check the asymmetry of funnel plot.

#### 2.5.3 Subgroup analysis

If the included studies have significant heterogeneity, subgroup analysis analysis will be used to find the source of heterogeneity.

#### 2.5.4 Sensitivity analysis

When sufficient studies are available, sensitivity analysis will be used to assess the robustness of the meta-analysis based on methodological quality, sample size, and missing data.

#### 2.5.5 Grading the quality of evidence

We will assess the quality of evidence by the Grading of Recommendations Assessment, Development and Evaluation and rate it into high, moderate, low or very low 4 levels.^[7,8]^

## 3 Discussion

Moving cupping therapy has the effect of cupping, scraping, massage and other treatment methods.^[9]^ Through the adsorption of the can on the skin, and sliding on the skin, it can play the role of warming the meridians, promoting blood circulation and removing blood stasis. At the same time, moving cupping therapy has the effect of opening and expelling pathogenic factors, strengthening the body and eliminating pathogenic factors.^[10]^

At the same time, other clinical studies have reported that moving cupping therapy can reduce the recurrence rate of psoriasis patients,^[11,12]^ reduce scales and plaque thinning, strengthen the absorption of topical drugs,^[13]^ and regulate the patient's autoimmunity.^[14]^ In conclusion, the efficacy of moving cupping therapy for the treatment of psoriasis vulgaris has certain advantages compared with other treatment methods, and it is easy to operate, economic and convenient, which is worthy of clinical application. However, there are few literatures and quality deviation in this study. Therefore, a large sample and multi center randomized controlled blind trial is needed to further confirm the effectiveness of cupping therapy in the treatment of psoriasis vulgaris, and to develop relatively effective cupping scheme, cupping medium and cupping manipulation, so as to be widely used in clinical practice and better serve patients.

## Author contributions

**Conceptualization:** Liu Mingqiang, Qiu Guirong.

**Data curation:** Liu Mingqiang, Qiu Guirong.

**Formal analysis:** Liu Mingqiang, LAN Hongrong, Huang Gang.

**Funding acquisition:** Liu Mingqiang.

**Investigation:** Wu Yunbo, LAN Hongrong.

**Methodology:** Wu Yunbo, Huang Gang.

**Project administration:** Qiu Guirong, Wu Yunbo.

**Resources:** Wu Yunbo.

**Software:** LAN Hongrong, Wu Yunbo.

**Supervision:** Wu Yunbo, Qiu Guirong.

**Validation**: Liu Mingqiang, Wu Yunbo.

**Visualization:** Liu Mingqiang, Wu Yunbo, Huang Gang.

**Writing – original draft:** Liu Mingqiang, Qiu Guirong.

**Writing – review & editing:** Liu Mingqiang, Qiu Guirong.

## Correction

The funding information originally published incorrectly as “JXSYLXK- ZHY1048” and has been corrected to “JXSYLXK-ZHY1047”.

## Abbreviations

Abbreviations: CI = confidence interval, ICTRP = International Clinical Trials Registration Platform, MD = mean difference, PASI = psoriasis area and severity index, RCT = randomized controlled trial, RR = risk ratio, SMD = standardized mean difference.
